# Diverting Stoma-Related Evisceration: A Comprehensive Review of 28 Case Reports Published in the Medical Literature in English

**DOI:** 10.7759/cureus.59621

**Published:** 2024-05-04

**Authors:** Ioan Nicolae Mateș

**Affiliations:** 1 Clinic of General and Esophageal Surgery, Saint Mary Clinical Hospital, Carol Davila University of Medicine and Pharmacy, Bucharest, ROU

**Keywords:** emergency surgery, colorectal surgery, transstomal evisceration, parastomal evisceration, stoma complications

## Abstract

Evisceration is an exceptional complication of diverting a stoma (a common procedure, often considered a minor surgery) with peculiar, specific, features (distinct-to-usual incisional evisceration), due to the presence of a stoma. Available data are limited to a few case reports; some aspects are not fully documented. The results of 28 case reports (full-text articles published in the English literature) were analyzed using 14 variables: age and gender; pathology; surgical setting; index surgery and type of stoma; intended stoma creation; time from surgery to evisceration; type of evisceration; visceral content; cause of evisceration; specific predisposing/risk factors; surgical approach; resection of nonviable content; surgical stoma treatment; and short-term outcome. Urgent surgery resulted in 46.42% resection of nonviable eviscerated content and 7.14% mortality.

All issues (some not discussed in previous reviews) were analyzed, to highlight their clinical relevance for surgical practice. The mechanisms (types of evisceration) are different in parastomal and transstomal/intrastomal evisceration; they should be considered as distinct entities. The real (underlying) etiology was identified in 26/28 case reports (92.85%): surgical failure, such as inadequate technique/tactics/strategy (12/26 case reports, 46.15%); trauma (7/26 case reports, 26.92%); and spontaneous necrosis (6/26 case reports, 21.42%). Parastomal hernia and/or prolapse (10/28 case reports, 35.71%) were specific predisposing factors; in such cases, early surgical treatment is recommended.

Temporary stoma was a potential risk factor, both for early as well as for late evisceration (e.g., long-standing temporary stoma); in such cases, early take-down or conversion to definitive stoma is beneficial. A local surgical approach (avoiding median laparotomy) was used in 13/28 (46.42%) of case reports. Seven different surgical options were used for surgical stoma treatment, demonstrating versatility; the initial stoma site was preserved in 22/28 (78.57%) of case reports.

## Introduction and background

Temporary/permanent diverting stoma (ileostomy/colostomy) is a common surgical procedure (often considered a minor surgery) but is associated with its own morbidity/mortality; complications are described as early (within 30 days after surgery) or late. Usual complications can be avoided through adherence to meticulous techniques and sound surgical principles [1]; elective take-down of temporary stoma can also be associated with morbidity/mortality [2]. The incidence of usual complications is a variable reported in a retrospective analysis [3], a multivariate analysis [4], a literature overview [5], a systematic review of selected randomized controlled trials (RCTs) [6], consensus guidelines [7], and a retrospective study [8].

Evisceration related to stoma formation is unusual (exceptional) and neglected in these studies; it is associated with a high rate of morbidity and even mortality (a potentially life-threatening condition; immediate surgery is mandatory) and is relatively undocumented in previous reviews. The objective of this review is to realize a synthesis of published case reports providing a comprehensive analysis of issues related to the topic (some not discussed in previous reviews) to highlight their clinical relevance for surgical practice and to offer information to the medical community.

## Review

Methods

A comprehensive search of academic databases, English literature (MEDLINE (Medical Literature Analysis and Retrieval System Online), NCBI (National Center for Biotechnology Information), PubMed Central, Clarivate, Web of Science; Google Scholar; and Google search), using ”parastomal evisceration” and ”transstomal evisceration” as keywords, revealed only 28 case reports, published in 27 full-text articles (2008-2023). Studies published in Portuguese [9,10] or Japanese [11], with abstracts in English (not full text) were excluded.

Results of each case report were briefly described (with minimal additional comments), to facilitate comparison of their key characteristics, in a tabular structure; 14 variables were studied (age and gender, pathology, surgical setting, index surgery, type of stoma, intended stoma creation, time from surgery to evisceration, type of evisceration and visceral content, cause of evisceration, specific predisposing/risk factors, surgical approach, resection of nonviable content, surgical ostomy treatment, and short-term outcomes). There were no twin case reports; each demonstrated individual characteristics. Specific statistical synthesis was virtually impossible for the following reasons: the absence of series, the limited number of patients (28 case reports); and underlying heterogeneity (14 variables), so a systematic review was not feasible.

Results

Studies were chronologically ordered and details of each case report are mentioned below.

Fitzgerald et al. [12], 2008, reported an early (after 10 days of surgery) parastomal small bowel (SB) evisceration in a 65-year-old male (M) after an anterior rectal resection (for rectosigmoid villous adenoma) with a defunctioning loop ileostomy. A high ileostomy output (on day six after index surgery, due to Clostridium difficile colitis) was followed by early ileostomy prolapse (that was manually reduced), and later on, by spontaneous mucocutaneous separation of ileostomy. A transverse parastomal incision was practiced, the content was reduced in the abdominal cavity, and the loop ileostomy was converted to end ileostomy. Although the abdominal wall opening appeared appropriately sized, the surgical failure was (probably) related to an inadequate fascia and mucocutaneous fixation; evisceration could have been prevented by surgical reduction of early prolapse.

Park et al. [13], 2010, reported the case of a 58-year-old male, with late (four months after surgery) parastomal SB evisceration after anterior rectal resection for rectal adenocarcinoma (ADK) with defunctioning loop ileostomy (complicated by a parastomal hernia and prolapse). Evisceration was iatrogenic: traumatic rupture (secondary to forceful manipulation to reduce prolapse). A resection of strangulated SB was realized, followed by ileostomy closure.

Carmen Nofuentes et al. [14], 2011, reported the case of an 83-year-old male (with a history of senile dementia) who was operated on for rectal ADK (abdominoperineal amputation). Three months after index surgery a late transstomal evisceration of large omentum (LO) was reported. Evisceration occurred through a defect in the colostomy wall and it was secondary to self-induced trauma: the patient had the habit of introducing his index in the stoma. A local surgery approach was used to perform resection of LO and suture of the colonic parietal defect.

Moffett and Younggren [15], 2011, published the case of a 23-year-old male with a history of Crohn’s disease and prior ileostomy; the initial procedure is not mentioned in the case report. The end ileostomy was complicated by a late SB parastomal evisceration, caused by blunt abdominal trauma, requiring laparotomy, reduction, and recreation (repositioning) of ileostomy to his left side.

Vornehm et al. [16], 2011, reported a late (two years after surgery) SB parastomal evisceration in a 66-year-old female with a long history of Crohn’s disease after Hartmann’s procedure for perforated diverticulitis. Extensive chronic parastomal pyoderma gangrenosum created a large fascial defect. Reduction and repositioning of colostomy in the left upper quadrant was the surgical solution in this case.

Salles et al. [17], 2011, reported the case of a 62-year-old male with rectal ADK; he was taking an oncologic neoadjuvant treatment. The patient was urgently submitted to loop transversostomy for acute bowel obstruction (ABO) decompression. His comorbidity was chronic pulmonary disease (COPD); a bronchospasm crisis with intermittent cough worsened his respiratory condition, requiring mechanical ventilation sup­port four days after surgery. During this event, he presented a parastomal SB and transverse colon (TC) evisceration. The patient was reoperated through the stoma: SB reduction, partial resection of microperforated TC, and conversion to end TC colostomy. The hole diameter of the stoma was larger than re­quired for TC extrusion due to the large distension observed in the episode of ABO.

Villa et al. [18], 2012, reported the case of a 69-year-old male with the previous therapy sequence: transverse loop colostomy (emergency treatment of obstructing rectal ADK); neoadjuvant radiochemotherapy (for five months); and finally, elective low anterior rectal resection, leaving the temporary colostomy in situ. The course of the low anterior rectal resection was uneventful, so the closure of the temporary stoma was scheduled for three months later; unfortunately, it was not realized because evisceration occurred first. A late (eight months after surgery) transstomal SB evisceration was secondary to postoperative ABO (SB obstruction by omentum band and adhesions). Surgery consisted of laparotomy, SB reduction, and suture of colonic wall perforation below the mucocutaneous plane. Evisceration could have been prevented by an earlier take-down of the colostomy.

Guner et al. [19], 2012, published the case of a 76-year-old male with Hartmann’s procedure for sigmoid necrosis (ischemic gangrenous colitis) complicated (11 months later) by a late SB transstomal evisceration, occuring through a spontaneous perforation (10 cm proximal to the end colostomy opening). Evisceration was caused by the recurrence of diffuse ischemia (ischemic colitis), requiring laparotomy followed by SB reduction, partial colon resection, and recreation of the end colostomy.

Azouz et al. [20], 2014, reported the case of a 69-year-old male operated on for necrotizing perianal fasciitis (soft tissue debridement and diverting end sigmoidostomy), followed by early (three days after surgery) strangulated SB parastomal evisceration. Reoperation consisted of laparotomy, resection of 100 cm of compromised SB, and repair of the parastomal defect. A stomatal aperture larger than the ideal size was the cause of evisceration.

Yucel et al. [21], 2014, reported a 62-year-old male who underwent Miles’s operation for rectal ADK; a parastomal hernia occurred eight months after surgery. One year after index surgery, the following sequence occurred: irreducible parastomal hernia (ABO of SB demonstrated by plain abdominal X-ray), prolapsed colon, rupture of hernia, and SB evisceration during the observation period. Surgery consisted of expansion of the stoma opening, SB reduction, and closure of the fascial defect. The rupture of the parastomal hernia could have been prevented by immediate surgery.

Ramly et al. [22], 2014, reported the case of an 81-year-old male who underwent total completion abdominal colectomy (for refractory Clostridium difficile colitis) and end ileostomy (reinforced with intraperitoneal mesh), complicated by early prolapse and early (nine days after surgery) strangulated parastomal ileostomy evisceration, requiring SB resection and recreation of ileostomy. A lot of associated risk factors were identified, but early prolapse was the real cause of evisceration, so a technical failure cannot be excluded.

Lolis et al. [23], 2015, reported the case of a 48-year-old female with stage IV rectal ADK and end-transverse colostomy for rectovaginal fistula, presenting a late (18 months after surgery) SB and LO transstomal evisceration (exteriorized through the end-transverse colostomy); chronic colostomy prolapse in conjunction with the underlying parastomal hernia caused necrosis of the colostomy wall. The surgical solution was right hemicolectomy and loop sigmoidostomy. Evisceration was labeled as parastomal, but, obviously, it was a transstomal one.

Salles et al. [24], 2016, published a second case of early (10 days after surgery) SB parastomal evisceration following loop transversostomy in an 82-year-old male, similar to a previous report [17]. Once again a parastomal approach was used for SB reduction and resizing of the aponeurotic aperture. Similar to the previous case report, it was considered that the diameter of the stoma remained notably superior to the necessity of transverse exteriorization, attributed to its large distension at the time of the ABO. The clinical outcome was unfavorable (acute inflammatory response syndrome, pneumonia, and acute renal failure requiring mechanical respiratory support); the patient's condition worsened and he died seven days later.

Arbra and Fann [25], 2017, reported a 90-year-old male with subtotal colectomy and end-ileostomy (for failed conservative management of Ogilvie syndrome) and early (seven days after surgery) SB parastomal evisceration, preceded by atrial arrhythmia and respiratory failure (with subsequent tracheostomy). Emergency surgery showed an appropriately sized myofascial aperture created during the initial end-ileostomy. The operation consisted of SB reduction and stoma revision.

Basnayake et al. [26], 2019, described a 51-year-old male with temporary loop sigmoidostomy (for extensive hidradenitis suppurativa of the perineum), parastomal hernia, and stoma prolapse (six weeks after surgery). The authors considered that ischemia and weakening of the underlying abdominal wall and the overlying skin caused a late (six months after surgery) SB parastomal evisceration. Surgical treatment consisted of reduction and resiting of the sigmoid colostomy.

Kulkarni et al. [27], 2019, highlighted the importance of meticulous surgical technique and emergency surgical intervention in two case reports of early parastomal eviscerations secondary to loop-diverting sigmoidostomy. Case 1 was a 45-year-old male operated on for repair of an iatrogenic rectal injury (during surgery for sacral chordoma). An early evisceration of TC and LO occurred on day 12 after surgery, preceded by a severe bout of coughing. The creation of a larger aperture to accommodate the dilated sigmoid (distended and loaded with hard stool) was identified as a possible cause of evisceration. Surgery included reduction and resizing of the enlarged stoma opening. Case 2 was a 50-year-old female with locally advanced cervix carcinoma (squamous cell carcinoma (SCC)) and loop-diverting sigmoidostomy for rectovaginal fistula. The evolution of the case was complicated by an early (day nine after surgery) evisceration of SB loops (that were contaminated with feces and edematous because of their proximity to the colostomy site); contamination induced severe sepsis with multiorgan dysfunction. Reoperation consisted of reduction of SB through an extension of the previous incision and stoma reconstruction. The patient died five days later due to sepsis and multiorgan dysfunction syndrome.

Mateș et al. [28], 2020, reported the case of an 84-year-old male admitted for a two-month history of chronic low bowel obstruction (CLBO) due to a locally advanced rectal ADK. Prior to neoadjuvant therapy, a diverting loop sigmoidostomy (with no previous colonic preparation) was practiced; the colon was loaded with hard stool, similar to Kurkarni et al. (case 1) [27]. Colostomy was maturated on day two but it was not functional on day three, resulting in early SB parastomal evisceration (immediate surgery using a parastomal approach: SB reduction and suture of fascial disruption). In the pathological context, paralytic ileus was blamed as the cause of evisceration, but in retrospect, maybe, evisceration could have been prevented by instant stoma maturation; a surgical failure.

Lapena-Rodriguez et al. [29], 2020, presented the case report of a 44-year-old male, with a consecutive surgical history of sigmoidectomy (for relapsing volvulus), anastomosis leak, emergency end colostomy, parastomal hernia, and stoma prolapse five months later. Laparotomy was used as a surgical approach for a late (13 months after surgery) SB transstomal evisceration. Intraoperatively, necrosis and perforation in the lateral wall of the prolapsed end colostomy were demonstrated as the point for SB evisceration. Surgical treatment consisted of reduction of SB and partial resection of perforated end colostomy followed by recreation of end colostomy. Etiology was similar to [23], misinterpreted in both studies as parastomal evisceration, but was in fact transstomal.

Basnayake et al. [30], 2021, reported a 53-year-old male (with a history of bronchial asthma) diagnosed with stage IV ADK of hepatic flexure, without previous intestinal obstruction (according to contrast-enhanced CT). He developed ACO during bowel preparation for colonoscopy, so an emergency trephine loop ileostomy was created. Early (day seven after surgery) SB parastomal evisceration was followed by laparotomy, SB resection, and ileostomy refashioning. On day five, he died due to subsequent pneumonia and worsening acute respiratory distress syndrome (ARDS). A technical failure was identified as the cause of evisceration: a larger stoma opening to accommodate the dilated SB (secondary to ABO) so that the resolution of intestinal edema may have created enough space for intestinal evisceration.

Cecire et al. [31], 2021, reported a 77-year-old male with multiple previous surgeries for Crohn’s disease: right hemicolectomy (for terminal ileal disease) and subsequent loop colostomy (for rectal stricture) that was converted to an end colostomy (due to stomal prolapse). During a recent admission, the patient presented an episode of SB obstruction (secondary to adhesions) that was managed nonoperatively; he was noted to have a reducible stoma prolapse of 10 cm and a parastomal hernia containing SB. A blunt force (trauma) was responsible for late SB transstomal evisceration: a large colonic perforation proximal to the stoma, through which the SB protruded into the colonic lumen and then through the colostomy, a perforation in the colon that was known to prolapse at times. Laparotomy was followed by reduction of SB, partial colonic resection, and refashioning of stoma.

Rockson et al. [32], 2021, published the case of a 56-year-old male who underwent surgery for a locally advanced rectal ADK after receiving total neoadjuvant therapy. Loop sigmoidostomy was practiced for CLBO decompression; ~5 liters of the ascitic liquid were suctioned during index surgery without placing intra-abdominal drains. An early (day 10 after surgery) parastomal evisceration (with strangulated LO) was treated using a peristomal incision. Both the necrotic LO and the protruding, prolapsed segment of the stoma were resected and a new proximal colostomy was then rematurated; this time, two drains were placed to collect ascites. Ascites (not a lack of technique) was blamed for evisceration, but it could have been prevented by the drainage of ascites during index surgery.

Momah et al. [33], 2021, reported a 35-year-old male with a significant history (AIDS, prior infections from several AIDS-defining organisms, Kaposi sarcoma, chemotherapy and steroid use for adrenal insufficiency, diabetes mellitus, and protein malnutrition). Index surgery was a diverting end colostomy for invasive anal SCC. Blunt abdominal trauma was the cause of a late (seven weeks after surgery) SC and LO parastomal evisceration, which was treated using a parastomal approach (partial resection and recreation of the stoma).

Gómez Contreras et al. [34], 2022, reported a 77-year-old male with sigmoidectomy and end colostomy (for occlusive colon ADK) and late (four years after surgery) SB transstomal evisceration (caused by blunt abdominal trauma). Surgery consisted of laparotomy, SB reduction, and suture of the defect.

Karadaghi et al. [35], 2022, reported the case of a 62-year-old male who previously underwent a sigmoidectomy and end colostomy for rectal ADK. The patient presented a long-standing parastomal hernia that was ruptured (seven years after surgery) due to a blunt abdominal trauma, with a secondary occurence of a late SB transstomal evisceration. Urgent surgery consisted of laparotomy, reduction of SB, and repair of the colostomy defect with interrupted sutures.

Murphy et al. [36], 2023, reported a 54-year-old male presenting with a large parastomal evisceration containing >100 cm of strangulated, non-viable SB. A loop ileostomy was formed one year prior at the time of an elective ileocecectomy for Crohn’s disease. Several hours later an extension of the stoma site was practiced, followed by midline laparotomy, SB resection, and conversion to end ileostomy in the left side of the abdomen. The patient developed pneumonia in the immediate post-operative period, so that cough in the preceding days was presumed as the cause of increased intraabdominal pressure. Data was insufficient to identify the etiology of evisceration in this case.

Iswan et al. [37], 2023, reported a 50-year-old male with loop ileostomy (for obstructing rectal ADK), complicated three months later by parastomal hernia containing SB (managed non-operatively in the context of neoadjuvant therapy). Rupture of the neglected parastomal hernia was, probably, the cause of the late (seven months after surgery) parastomal evisceration. The patient was reoperated using a parastomal approach, SB reduction, and stoma revision.

Hasnaoui et al. [38], 2023, reported the case of a 58-year-old female, operated on for perforated cecal ADK (generalized purulent peritonitis): right colectomy and end-loop ileocolostomy (double-barrel stoma), followed by six sessions of adjuvant chemotherapy. A suspicion of peritoneal carcinomatosis (based on CT scans and abdominal MRI) was confirmed (frozen section report) during an elective reoperation (laparotomy) one month later; stoma mobilization was unable to offer a comprehensive peritoneal cavity exploration, thus a midline incision was executed and the decision was made to retain the double-barrel stoma. Early (two days after surgery) parastomal evisceration (1 m of viable SB), in the context of ongoing paralytic ileus, was determined by a surgical faiure: a large defect in the musculoaponeurotic sheath and consequent mucocutaneous dehiscence at the inferior angle of the stoma site. Immediate emergency surgery was practiced using a parastomal approach: SB reduction and stoma revision.

Discussion

Previous reviews [27,28,30,31,36-38] included fewer case reports and variables. Issues related to the topic (some not discussed before) are described and analyzed to highlight their clinical relevance for surgical practice. Results are summarized in Tables 1, 2.

**Table 1:** Summary of case reports M: male; F: female; ADK: adenocarcinoma; SCC: squamous cell carcinoma; ABO: acute bowel obstruction; CLBO: chronic low bowel obstruction; E: elective surgery; U: urgent surgery

Author, reference, year	Age, gender	Pathology	Surgical setting	Index surgery; type of stoma	Intended stoma creation	Time from surgery to evisceration
Fitzgerald et al. [12], 2008	65 M	Rectosigmoid villous adenoma	E	Anterior rectal resection; loop ileostomy	Temporary; defunctioning	Early, 10 days
Park et al. [13], 2010	58 M	Rectal ADK	E	Anterior rectal resection; loop ileostomy	Temporary; defunctioning	Late, four months
Carmen Nofuentes et al. [14], 2011	83 M	Rectal ADK	E	Miles; end colostomy	Permanent	Late, three months
Moffett and Younggren [15], 2011	23 M	Crohn’s disease	E	End ileostomy	Permanent	Late, not specified
Vornehm et al. [16], 2011	66 F	Perforated diverticulitis	U	Hartmann; end colostomy	Permanent	Late, two years
Salles et al. [17], 2011	62 M	Rectal ADK; ABO	U	Loop transverostomy	Temporary; decompression	Early, four days
Villa et al. [18], 2012	69 M	Rectal ADK	E	Anterior rectal resection; loop transversostomy	Temporary; left in situ	Late, eight months
Guner et al. [19], 2012	76 M	Ischemic gangrenous colitis	U	Hartmann; end colostomy	Permanent	Late, 11 months
Azouz et al. [20], 2014	69 M	Necrotizing perianal fasciitis	U	Debridement; end colostomy	Temporaray; diverting	Early, three days
Yucel et al. [21], 2014	62 M	Rectal ADK	E	Miles; end colostomy	Permanent	Late, one year
Ramly et al. [22], 2014	81 M	Clostridium difficile colitis	U	Completion of colectomy; end ileostomy	permanent	Early, nine days
Lolis et al. [23], 2015	48 F	Rectal ADK; rectovaginal fistula	E	End transversostomy	Permanent; diverting	Late, 18 months
Salles et al. [24], 2016	82 M	Rectal ADK; ABO	U	Loop transversostomy	Temporary; decompression	Early, 10 days
Arbra and Fann [25], 2017	90 M	Ogilvie syndrome	U	Subtotal colectomy; end ileostomy	Permanent	Early, seven days
Basnayake et al. [26], 2019	51 M	Perineal suppurative hidradenites	U	Loop sigmoidostomy	Temporary; diverting	Late, six months
Kulkarni et al. [27], 2019 (case 1)	45 M	Sacral chordoma, iatrogenic rectal injury	E	Loop sigmoidostomy	Temporary; diverting	Early, 12 days
Kulkarni et al. [27], 2019 (case 2)	50 F	Cervix SCC; rectovaginal fistula	E	Loop sigmoidostomy	Permanent; diverting	Early, nine days
Mates et al. [28], 2020	84 M	Rectal ADK; CLBO	E	Loop sigmoidostomy	Temporary; diverting	Early, three days
Lapena-Rodriguez et al. [29], 2020	44 M	Sigmoid volvulus	U	Sigmoidectomy; end colostomy	Temporary; diverting	Late, 13 months
Basnayake et al. [30], 2021	53 M	Colonic ADK; iatrogenic ABO	U	Loop ileostomy	Temporary; decompression	Early, seven days
Cecire et al. [31], 2021	77 M	Crohn’s disease	E	End colostomy	Permanent	Late, not specified
Rockson et al. [32], 2021	56 M	Rectal ADK; CLBO	E	Loop sigmoidostomy	Temporary; decompression	Early, 10 days
Momah et al. [33], 2021	35 M	Anal SCC; perianal fistula	E	End colostomy	Permanent; diverting	Late, two months
Gómez Contreras et al. [34], 2022	77 M	Colonic ADK	E	Sigmoidectomy; end colostomy	Permanent	Late, four years
Karadaghi et al. [35], 2022	62 M	Rectal ADK	E	Sigmoidectomy; end colostomy	Permanent	Late, seven years
Murphy et al. [36], 2023	54 M	Crohn’s disease	E	Ileocecectomy; loop ileostomy	Permanent	Late, one year
Iswan et al. [37], 2023	50 M	Rectal ADK; CLBO	E	Loop ileostomy	Temporary; defunctioning	Late, seven months
Hasnaoui et al. [38], 2023	58 F	Perforated cecal ADK	E	Right colectomy; loop ileostomy; explorative laparotomy	Temporary; defunctioning	Early, two days

**Table 2:** Summary of case reports P: parastomal; T: transstomal; SB: small bowel; TC: transverse colon; SC: sigmoid colon; LO: large omentum; NI: none identified; L: laparotomy; P: parastomal; T: transstomal; F: favorable; D: deceased

Author, reference, year	Type of evisceration	Visceral content	Cause	Specific predisposing/risk factors	Surgical approach	Resection of nonviable content	Surgical stoma treatment	Short-term outcome
Fitzgerald et al. [12], 2008	P	SB	Surgical failure; tactics	Early loop ileostomy prolapse	P	-	Conversion to end ileostomy	F
Park et al. [13], 2010	P	SB	Traumatic; iatrogenic	Parastomal hernia and prolapse	L	SB	Ileostomy take-down	F
Carmen Nofuentes et al. [14], 2011	T	LO	Traumatic; self-induced	NI	T	LO	Repair of transstomal defect	
Moffett and Younggren [15], 2011	P	SB	Traumatic; accidental	NI	L	-	Repositioning of stoma	F
Vornehm et al. [16], 2011	P	SB	Necrosis	Pyoderma gangrenosum	L	-	Repositioning of stoma	F
Salles et al. [17], 2011	P	SB, TC	Surgical failure; technical	Respiratory failure (mechanical ventilation)	P	TC	Conversion to end colostomy	F
Villa et al. [18], 2012	T	SB	Postoperative ABO of SB	Omentum band, adhesions	L	-	Repair of transstomal defect	F
Guner et al. [19], 2012	T	SB	Necrosis	Ischemic colitis	L	SC	Partial stoma resection, recreation of stoma	F
Azouz et al. [20], 2014	P	SB	Surgical failure; technical	NI	L	SB	Stoma revision	F
Yucel et al. [21], 2014	P	SB	Surgical failure; strategy	Parastomal hernia and prolapse	P	-	Stoma revision	F
Ramly et al. [22], 2014	P	SB	Surgical failure; technical	Early end ileostomy prolapse	P	SB	Stoma revision	F
Lolis et al. [23], 2015	T	SB, LO	Necrosis	Parastomal hernia and prolapse	L	TC	Right hemicolectomy, loop sigmoidostomy	F
Salles et al. [24], 2016	P	SB	Surgical failure; technical	Respiratory failure (mechanical ventilation)	P	-	Stoma revision	D
Arbra and Fann [25], 2017	P	SB	NI	Respiratory failure (mechanical ventilation	L	-	Stoma revision	F
Basnayake et al. [26], 2019	P	SB	Necrosis	Parastomal hernia and prolapse	L	-	Repositioning of stoma	F
Kulkarni et al. [27], 2019 (case 1)	P	TC, LO	Surgical failure; technical	NI	P	-	Stoma revision	F
Kulkarni et al. [27], 2019 (case 2)	P	SB	Surgical failure; technical	NI	P	-	Stoma revision	D
Mates et al. [28], 2020	P	SB	Surgical failure; strategy	Postoperative paralytic ileus	P	-	Stoma revision	F
Lapena-Rodriguez et al. [29], 2020	T	SB	Necrosis	Parastomal hernia and prolapse	L	SC	Partial stoma resection, recreation of stoma	F
Basnayake et al. [30], 2021	P	SB	Surgical failure; technical	NI	L	SB	Stoma revision	D
Cecire et al. [31], 2021	T	SB	Traumatic; accidental	Parastomal hernia and prolapse	L	SC	Partial stoma resection, recreation of stoma	F
Rockson et al. [32], 2021	P	LO	Surgical failure; strategy	NI	P	LO, SC	Partial stoma resection, recreation of stoma	F
Momah et al. [33], 2021	P	LO, SC	Traumatic; accidental	AIDS, immunosuppression	P	SC	Partial stoma resection, recreation of stoma	F
Contreras et al. [34], 2022	T	SB	Traumatic; accidental	NI	L	-	Repair of transstomal defect	F
Karadaghi et al. [35], 2022	T	SB	Traumatic; accidental	Parastomal hernia	L	-	Repair of transstomal defect	F
Murphy et al. [36], 2023	P	SB	NI	NI	L	SB	Repositioning of stoma	F
Iswan et al. [37], 2023	P	SB	Necrosis	Parastomal hernia	P	-	Stoma revision	F
Hasnaoui et al. [38], 2023	P	SB	Surgical failure; technical	Postoperative paralytic ileus	P	-	Stoma revision	F

Age and Gender

The mean age (all patients) was 61.78 years: higher in males (62.83 years; range 23 to 90 years) compared to females (55.5 years). Advanced age and male dominance (85.71%) were not considered risk factors; their significance is related to pathology.

Pathology

Malignancy (16/28, 57.14%) was not demonstrated as a predisposing factor. Rectal ADK (12/16, 75%) was the main indication of malignancy, but in most instances, evisceration was not related to the malignant condition itself but to the temporary diversion: ileostomy [13,37] or transversostomy [17,18,23,24].

Surgical Setting

Emergency surgery was not a predisposing factor; 64.28% of eviscerations occurred after elective surgery.

Index Surgery and Type of Stoma

Both ileostomy and colostomy eviscerations were studied. Data regarding ileostomy (32.14%) was limited; this review did not include a comparison of ileostomy vs. colostomy evisceration. The occurrence after loop ileostomy/colostomy (53.57%) was similar to end-ostomy; the type of stoma was irrelevant.

Intended Stoma Creation

Incidences after permanent and temporary stomas were identical (50%); they must be discussed according to the time after the creation of the stoma.

Time From Surgery to Evisceration

Similar to usual stoma complications, evisceration was defined as early (within 30 days after surgery) or late (after that interval). Early eviscerations (2-12 days, mean 7.16 days) were reported in 12/28 (42.85%) case reports, mainly associated with temporary stomas [12,17,20,24,27,28,30,32]. The risk accumulates in time so that most (16/28, 57.14%) were late eviscerations (2-84 months, mean 18 months; interval not reported in two case reports), including long-standing temporary stomas [13,18,26,29,37]. Temporary stomas were a potential risk factor for both early and late eviscerations, suggesting the utility of early take-down or conversion to a permanent ostomy.

Type (Mechanism) of Evisceration

Parastomal and transstomal eviscerations are distinct entities as far as the site of evisceration in relation to the exteriorized viscus, time from surgery, and surgical stoma treatment are concerned. Parastomal evisceration (20/28, 71.42%) appeared alongside the viscus, both in early [12,17,20,22,24,25,27,28,30,32,38] and late eviscerations [13,15,16,21,26,33,36,37]. Transstomal/intrastromal evisceration (8/28, 28.57%) was always a late one (range: three months to seven years, mean 26.43 months), as the result of a condition that perforates the stoma wall permitting evisceration within its lumen. The term “evisceration through the stoma” is confusing, a misnomer; transstomal colostomy evisceration was misinterpreted as parastomal in two case reports [23,29].

Visceral Content

The eviscerated content varied according to each case report and it was not connected to the type of stoma or mechanism of evisceration: SB (24/28; 85.71%), TC [17,27], SC [33], LO alongside a hollow viscus [23,27,33], or only LO [14,32].

Cause of Evisceration

Knowledge of etiology is paramount for prevention. Previous studies [13,14,17-25,27,28,30,36-38] focused on alleged predisposing risk factors: COPD, cough in the postoperative period; increased intra-abdominal pressure; chronic alcohol abuse, smoking; emergency surgery; advanced age; disseminated cancer, ascites; poor nutrition; immunosuppressive therapy, HIV; and diabetes mellitus. Risk factors (e.g., comorbidities) are associated with any incisional evisceration and are not specific to stoma evisceration; they can not be responsible if a direct, specific, cause is identified. The real (underlying) etiology was not discussed in previous reviews. Three major factors were identified in 26/28 case reports (92.85%). The first factor was a surgical failure (12/26, 46.15%), an inadequate technique such as the construction of a larger than optimal size parietal breach followed by early evisceration [17,20,22,24,27,30,38], or inadequate tactics/strategy [12,21,28,32]. second, a traumatic event was the cause of late evisceration (7/26, 26.92%); three case reports [13,31,35] were associated with traumatic rupture of parastomal hernia/prolapse. Pros [35] and cons [31] were expressed about the utility of early stoma revision, to prevent accidental rupture in case of blunt abdominal trauma. The third factor was spontaneous necrosis, mainly in late eviscerations (6/26, 21.42%), associated with the erosion of parastomal hernia/prolapse [23,26,29,36], pyoderma gangrenosum [6], or ischemic colitis [19].

Specific (Associated) Risk Factors

A specific risk factor was considered only if directly related to the cause of stoma evisceration. Parastomal hernia and/or prolapse (10/28, 35.71%) were specific predisposing factors both for early [12,22] and late [13,21,23,26,29,31,35,37] stoma evisceration; their presence was a determining factor (evisceration could not happen in their absence); early surgical treatment is recommended to prevent stoma evisceration. Respiratory failure (mechanical ventilation) [17,24,25] was an associated, but not a specific risk factor.

Surgical Approach

Surgical access in relation to time from surgery was not discussed in previous reviews. The presence of stoma permitted three different types. First: laparotomy (53.57%), especially for late [13,15,16,18,19,23,26,29,31,34-36] rather than for early evisceration [20,25,30]. Second: parastomal approach (42.85%), especially for early [12,17,22,24,27,28,32,37,38] rather than for late evisceration [21,33]. Third: transstomal approach for a late evisceration [14]. A local surgical approach (avoiding laparotomy) was used in 13/28 (46.42%).

Resection of Nonviable Content

Urgent surgery was practiced in all case reports; however, resection of eviscerated content of the evisceration was necessary in 13/28 (46.42%), including compromised (nonviable) SB [13,20,22,30,36], SC [19,29,31-33], TC [17,23], or LO [14,32].

Surgical Stoma Treatment

Seven different options were used for surgical stoma treatment, demonstrating a high versatility: stoma revision/refashion [20-22,24,25,27,28,30,37]; partial resection and recreation of initial stoma [19,29,31-33]; repair of tansstomal defect [14,18,34,35]; repositioning to a new site [15,16,23,36]; conversion to another type of stoma in the same location [12,17]; take-down of temporary ileostomy [13]; and stoma resection [23]. The initial stoma site was preserved in 22/28 case reports (78.57%).

Short-Term Outcomes

A favorable outcome (up to 30 days after urgent surgery for stoma evisceration) was reported in 26/28 case reports (92.85%), with only two fatalities [24,27].

## Conclusions

Diverting stoma-related evisceration is an exceptional complication of a common surgical procedure, relatively undocumented but associated with high morbidity and mortality in spite of immediate surgery. A number of issues related to the topic (including those not discussed in previous reviews) were analyzed to highlight their clinical relevance for surgical practice. Some (e.g., age and gender, malignancy, emergency surgery, type of stoma, intended stoma creation, and time from surgery to evisceration and visceral content) were irrelevant in this review.

Parastomal and transstomal eviscerations are distinct entities. Etiology includes surgical failure, trauma, and spontaneous necrosis. A temporary stoma is a potential risk factor for early evisceration and also for late evisceration (e.g., long-standing temporary ostomy), suggesting the utility of early takedown or conversion to a permanent stoma. Parastomal hernia and/or prolapse were specific predisposing factors; early surgical treatment is recommended. Unlike common incisional evisceration, stoma evisceration is characterized by variability in selecting the proper surgical options. Three different types of surgical approach were practiced; a local access (avoiding laparotomy) was used in 13/28 cases (46.42%). There were seven options for surgical stoma treatment; the initial site of the stoma was preserved in 22/28 case reports (78.57).

## References

[1] Kwiatt M, Kawata M (2013). Avoidance and management of stomal complications. Clin Colon Rectal Surg.

[2] Fonseca AZ, Uramoto E, Santos-Rosa OM, Santin S, Ribeiro M Jr (2017). Colostomy closure: risk factors for complications. Arq Bras Cir Dig.

[3] Park JJ, Del Pino A, Orsay CP, Nelson RL, Pearl RK, Cintron JR, Abcarian H (1999). Stoma complications: the Cook County Hospital experience. Dis Colon Rectum.

[4] Duchesne JC, Wang YZ, Weintraub SL, Boyle M, Hunt JP (2002). Stoma complications: a multivariate analysis. Am Surg.

[5] Shabbir J, Britton DC (2010). Stoma complications: a literature overview. Colorectal Dis.

[6] Malik T, Lee MJ, Harikrishnan AB (2018). The incidence of stoma related morbidity - a systematic review of randomised controlled trials. Ann R Coll Surg Engl.

[7] Miller AS, Boyce K, Box B (2021). The Association of Coloproctology of Great Britain and Ireland consensus guidelines in emergency colorectal surgery. Colorectal Dis.

[8] Benedek Z, Kocsis L, Bauer O (2022). Stoma-related complications: a single center experience and literature review. J Interdiscip Med.

[9] Corsi PR, Lanterno G, Pereira CS, Rasslan S (1991). Evisceracao transcolostomia: relato de caso [Article in Portuguese]. Revista brasileira de colo-proctologia.

[10] Solarana OJ, Rodríguez PY, Suárez LC, Pérez PA (2019). Transstomal small bowel evisceration after colonic perforation secondary to typhoid fever. A case purpose [Article in Portuguese]. Correo Científico Médico.

[11] Ito E, Ohira H, Saito T (2015). A case of prolapse of the small intestine due to opening of the colostomy route [Article in Japanese]. Nihon Gekakei Rengo Gakkaishi.

[12] Fitzgerald JE, Tang SW, Lake EJ, Richards T, Acheson AG (2008). Small bowel evisceration: a rare complication of laparoscopic ileostomy. Colorectal Dis.

[13] Park SJ, Lee SH, Lee KY (2010). Small bowel evisceration by rupture of prolapsed loop ileostomy. Colorectal Dis.

[14] Carmen Nofuentes R, Mario Mella L, Susana Perez B, Edelmira Soliveres S, Andres Garcia M, Salvador Garcia G (2011). Transstomal evisceration: a rare complication of colostomies. Rev Chil Cir.

[15] Moffett PM, Younggren BN (2010). Parastomal intestinal evisceration. West J Emerg Med.

[16] Vornehm ND, Kelley SR, Ellis BJ (2011). Parastomal small bowel evisceration as a result of parastomal pyoderma gangrenosum in a patient with Crohn's disease. Am Surg.

[17] Salles VJ, Saba E, Pissinin ER, Arguello ER, Filho HN (2011). Complication related to colostomy orifice: intestinal evisceration. J Coloproctol.

[18] Villa M, Iannelli E, Grande M, Rossi P, Tucci G (2012). An unusual case of small intestine evisceration through a transverse loop colostomy. Colorectal Dis.

[19] Guner A, Kahraman I, Ozkan OF, Aktas A, Kece C (2012). Transstomal small bowel evisceration after colonic perforation secondary to ischemic colitis. Case Rep Surg.

[20] Azouz V, Simmons JD, Abourjaily GS (2014). Immediate postoperative parastomal end sigmoid hernia resulting in evisceration and strangulation. J Surg Case Rep.

[21] Yucel AF, Pergel A, Aydin I, Sahin DA (2014). A rare stoma-related complication: parastomal evisceration. Indian J Surg.

[22] Ramly EP, Crosslin T, Orkin B, Popowich D (2016). Strangulated ileostomy evisceration following lateralizing mesh repair of parastomal hernia. Hernia.

[23] Lolis ED, Savvidou P, Vardas K, Loutseti D, Koutsoumpas V (2015). Parastomal evisceration as an extremely rare complication of a common procedure. Ann R Coll Surg Engl.

[24] Salles VJ, Mardegan C, Pereira MJ (2016). Intestinal extrusion by the hole of colostomy. World J Res Rev.

[25] Arbra CA, Fann SA (2017). Parastomal evisceration: rare complication after total abdominal colectomy. Am Surg.

[26] Basnayake O, Jayarajah U, Jayasinghe J, Wijerathne PK, Samarasekera DN (2019). Spontaneous rupture of a parastomal hernia with evisceration of small bowel: a case report. BMC Surg.

[27] Kulkarni AA, Chauhan V, Sharma V, Singh H (2019). Parastomal evisceration: a report of two cases and review of literature. Cureus.

[28] Mateş IN, Gheorghe M, Tomşa R, Sumedrea EL (2020). Paracolostomy evisceration: short review and a new case report. Chirurgia (Bucur).

[29] Lapeña-Rodríguez M, Fernández-Moreno MC, Martinez-Montava E, Muñoz-Forner E, Ortega J (2020). Late parastomal evisceration. Int J Colorectal Dis.

[30] Basnayake O, Prasanthan Y, Jayarajah U, Ganga N, De Silva K (2021). Early parastomal evisceration of small bowel following a loop ileostomy for malignant intestinal obstruction. SAGE Open Med Case Rep.

[31] Cecire J, Sarkar A, Ross W, Sutherland A (2021). Colonic perforation and transstomal evisceration of small bowel. J Curr Surg.

[32] Rockson O, Mhand M, Aabdi H, Bouhout T, El Harroudi T, Serji B (2021). Colostomy orifice complications: a case report of a prolapsed colostomy with necrosis of the eviscerated greater omentum. J Surg Case Rep.

[33] Momah U, Barnaby J, Poulos C, Lewis R (2021). Traumatic colostomy evisceration in an AIDS patient with anal cancer. J Surg Case Rep.

[34] Contreras RG, Navarro EM, Aicart AI, Martín JJ (2023). Transstomal evisceration due to blunt abdominal trauma. Cir Esp (Engl Ed).

[35] Karadaghi RL, Moschidis T, Zaheer M, Huttman M, Klein M (2022). A rare presentation of intra-stomal small bowel evisceration secondary to blunt trauma to a pre-existing parastomal hernia. Ann Clin Case Rep.

[36] Murphy AE, Stakelum A, Razzak S, Ashraf J, Awan F (2023). An extremely rare complication of intestinal stoma formation: parastomal evisceration 1-year post-loop ileostomy. J Surg Case Rep.

[37] Izwan S, Perera OM, Guy S (2023). Late parastomal evisceration: a case report of a rare complication following loop ileostomy for an obstructing rectal cancer. Int J Surg Case Rep.

[38] Hasnaoui A, Trigui R, Heni S, Kacem S (2023). Early postoperative parastomal evisceration after explorative laparotomy: case report of a rare and potentially life-threatening surgical complication. Patient Saf Surg.

